# Parkinson’s disease detection based on features refinement through L1 regularized SVM and deep neural network

**DOI:** 10.1038/s41598-024-51600-y

**Published:** 2024-01-16

**Authors:** Liaqat Ali, Ashir Javeed, Adeeb Noor, Hafiz Tayyab Rauf, Seifedine Kadry, Amir H. Gandomi

**Affiliations:** 1 Department of Electrical Engineering, University of Science and Technology Bannu, Bannu, Pakistan; 2 Aging Research Center, Karolinska Institutet, Solna, Sweden; 3 Department of Information Technology, Faculty of Computing and Information Technology, King Abdulaziz University, 80221 Jeddah, Saudi Arabia; 4 Bool Mind Software Technologies, Mequon, WI 53092 USA; 5 Department of Applied Data Science, Noroff University College, Kristiansand, Norway; 6 Artificial Intelligence Research Center (AIRC), Ajman University, Ajman, 346 United Arab Emirates; 7 Department of Electrical and Computer Engineering, Lebanese American University, Byblos, Lebanon; 8 Faculty of Engineering and Information Technology, University of Technology Sydney, Ultimo, NSW 2007 Australia; 9 University Research and Innovation Center (EKIK), Óbuda University, Budapest, 1034 Hungary

**Keywords:** Computational science, Information technology, Data mining, Data processing, Machine learning

## Abstract

In previous studies, replicated and multiple types of speech data have been used for Parkinson’s disease (PD) detection. However, two main problems in these studies are lower PD detection accuracy and inappropriate validation methodologies leading to unreliable results. This study discusses the effects of inappropriate validation methodologies used in previous studies and highlights the use of appropriate alternative validation methods that would ensure generalization. To enhance PD detection accuracy, we propose a two-stage diagnostic system that refines the extracted set of features through $L_{1}$ regularized linear support vector machine and classifies the refined subset of features through a deep neural network. To rigorously evaluate the effectiveness of the proposed diagnostic system, experiments are performed on two different voice recording-based benchmark datasets. For both datasets, the proposed diagnostic system achieves 100% accuracy under leave-one-subject-out (LOSO) cross-validation (CV) and 97.5% accuracy under k-fold CV. The results show that the proposed system outperforms the existing methods regarding PD detection accuracy. The results suggest that the proposed diagnostic system is essential to improving non-invasive diagnostic decision support in PD.

## Introduction

Parkinson’s disease (PD) is a neurological condition characterized by slowness of movements, tremors, rigidity, impaired voice and challenges in maintaining balance and coordination^1–3^. Global estimates in 2019 showed over 8.5 million individuals with PD^4^. In 1817 Dr. James Parkinson described and named the disease^5^. Speech-related impairments identified in PD patients include hypophonia (low volume), monotone speech (unvaried pitch range), dysarthria (difficulty in controlling speech-producing muscles), and dysphonia (difficulty in speaking)^6,7^. Approximately 90% of PD patients experience issues with their vocal system^6,8^. As of now, no medical (blood or laboratory) tests have been discovered for diagnosing PD^9,10^. Hence, artificial intelligence based methods using voice or speech features can facilitate neurologists.

The literature demonstrates that many machine learning methods have been introduced, utilizing voice and speech data, for the detection of PD^1,11^. Little et al. conducted an analysis of PD by measuring dysphonia^10^. Their dataset consisted of voice recordings from 31 individuals producing the vowel sound “a”. Dysphonia features were extracted from vowel phonation data and subsequently classified using the support vector machine (SVM) model. Tsanas et al. similarly employed voice data for the classification of PD^12^. A total of one hundred and thirty-two dysphonia measures were extracted from a dataset consisting of 263 samples^12^. Four feature selection algorithms were investigated to attain elevated accuracy. Huseyin Guruler utilized the dataset gathered in^10^ and accomplished the highest accuracy of 99.52% by employing a complex-valued artificial neural network with feature weighting based on k-means clustering^13^. Nonetheless, subject overlap emerged as a primary problem in Huseyin Guruler’s approach and other methods employed with the dataset from^10^. Furthermore, the preceding studies did not implement measures to mitigate the impacts of imbalanced classes within the dataset.

Sarkar et al.^6^ collected a well-balanced dataset from 20 PD patients and 20 healthy individuals to mitigate the influences of imbalanced classes distribution within the data. Each participant contributed twenty-six speech samples, and Praat acoustic analysis software was employed to extract 26 features from each speech sample^14^. Various learning models, including k-nearest neighbors (k-NN) and support vector machines (SVM), were investigated to attain optimal performance. However, the primary limitation for the well-balanced dataset obtained from^6^ was the comparatively lower classification accuracy. Canturk et al. aimed to enhance classification accuracy by employing a cascading approach, incorporating six distinct machine learning predictive models coupled with diverse feature selection algorithms. Nevertheless, their achieved maximum accuracies were 57.5% through Leave-One-Subject-Out Cross-Validation (LOSO CV) and 68.94% via 10-fold Cross-Validation (10-fold CV)^15^. Likewise, in a similar vein, ^16,17^, and^18^ compiled voice datasets with the intention of detecting PD. However, the datasets they employed are not accessible to the public. In reference to^16^, speech data from 50 subjects was collected. This study integrated three distinct feature extraction methods with five diverse classifiers, resulting in an impressive accuracy of 90%. In the context of^17^, a novel Bayesian linear regression technique was introduced for monitoring the severity of Parkinson’s Disease (PD) symptoms. This approach achieved an accuracy of 86.2% through the utilization of a two-stage variable selection and classification methodology.

Several researchers have explored deep learning models for PD diagnosis utilizing voice data, including techniques like autoencoders and Convolutional Neural Networks (CNNs)^19–21^. Several other scholars studied neural networks, but their study was limited to a single hidden layer, i.e., deep architecture was not explored^15,22,23^. Neural networks are commonly classified into two main categories: shallow neural networks (SNNs) and deep neural networks (DNNs). Shallow neural networks encompass an input layer, an output layer, and typically include only one hidden layer^24,25^. However, DNNs are characterized by an arrangement that comprises an input layer, an output layer, and multiple hidden layers^26,27^. In summary, DNNs are networks that undergo training using novel optimization algorithms and are composed of multiple hidden layers^28,29^. This study employs a recently introduced algorithm, namely the Adaptive Moment Estimation (ADAM) learning algorithm, for training the DNNs^30^.

This paper addresses two critical issues in PD detection using replicated voice and multiple types of speech data: the problem of inappropriate validation methods leading to subject overlap and a low rate of PD detection accuracy. Conventional k-fold CV is the cause of subject overlap. In such cases, we cannot depend on the constructed model as it is biased. Therefore, we suggest the use of alternative validation methodologies, such as LOSO CV. Additionally, we demonstrate that translating multiple samples per subject data into one sample per subject data automatically eliminates subject overlap.

To mitigate the low rate of PD detection accuracy problem, we have devised a two-stage diagnostic method to enhance PD detection accuracy. In the initial stage, we employ an $L_{1}$ regularized SVM model to refine the extracted features. Subsequently, in the following stage, we conduct classification using a DNN model. Different from previous work, we propose simultaneous optimization of the two models. To simultaneously optimize the two models, a hybrid grid is obtained by merging the hyper-parameters of the cascaded models. Optimized versions of SVM and DNN are constructed when the optimum point on the hybrid grid is identified. Hybrid grid search algorithm (HGSA)^31^ is used to locate the optimal point on the hybrid grid. The search algorithm can simultaneously optimize the two models, i.e., SVM and DNN. An optimum subset of features will be obtained through the optimized version of the SVM model, while the optimized version of DNN will work efficiently on an optimal subset of features.

The primary contributions of this paper can be succinctly summarized as follows:

1. This paper addresses the issue of inappropriate validation methods employed in prior studies and advocates for the adoption of alternative validation approaches. Furthermore, it demonstrates that consolidating multiple samples per subject data into a single sample per subject data set effectively mitigates the issue of overlap.
2. We enhance the set of extracted features through the utilization of an $L_{1}$-regularized SVM. This process effectively eliminates redundant and irrelevant features, yielding a higher-quality feature set for classification.
3. To the best of our knowledge, the proposed cascaded diagnostic system, referred to as $L_{1}$SVM-DNN, represents a pioneering technique for the detection of Parkinson’s disease (PD) using voice and speech data.
4. Only a limited number of studies have explored the evaluation of feature selection at the input level of Deep Neural Networks (DNN)^32^. Notably, Taherkhani et al.^32^ recently discovered that deep learning models exhibit improved performance when the feature selection and feature extraction capabilities of a DNN are integrated. In this paper, we reinforce this finding by incorporating feature selection at the input level of the DNN.
5. The proposed cascaded diagnostic system surpasses the performance of state-of-the-art methods as reported in the two benchmark voice recording datasets.

The remainder of the paper is structured as follows:

In Section “Materials and methods”, we provide a detailed explanation of the datasets and delve into the discussion of a deep learning-based predictive classification model. In Section “Results and discussion”, we present experimental results and engage in a discussion of these findings. Section “Comparative study” is dedicated to a comparative study. Section “Limitations of the study” briefly discuss some limitation of the study. Lastly, Section “Conclusion” encapsulates the conclusion of this study.

## Materials and methods

### Datasets description

Two datasets are used in this work. Max Little collected the first dataset in^10^ and is available at^33^. The second dataset was collected by Sarkar et al., reported in^6^ and can be obtained online from^34^. The Max Little (first dataset) data contains voice samples of 31 people (23 PD and eight healthy). The age range of the subjects is from 46 to 85 years (mean= $\mu =$ 65.8, std. deviation= $\sigma =$ 9.8). The duration of the disease for PD patients in the first dataset ranges from 1 to 28 years. The dataset contains 195 replicated sustained vowel “ a”   phonations. The data is a matrix containing 195 rows and 23 columns where the columns denote features except the last label column. The label can have a value of 0 or 1. A detailed description of 22 biomedical voice features extracted from each sample is given in Table 1.

The second dataset contains 20 healthy persons and 20 PD having PD for 0 to 6 years. Twenty-six voice samples, including words, numbers, sustained vowels, and short phrases, were taped for every individual. Praat acoustic analysis software was used to extract 26 features from every single voice sample^14^. A detailed description of these 26 features extracted from each sample is given in Table 1. Thus a total of 1040 samples are obtained. This data set is known as the training dataset. Another independent testing dataset was collected from 28 PD patients under the same conditions. This dataset was named the test dataset; it includes 168 samples. These samples include the recordings of 28 PD subjects, just saying vowels   “ a”   and   “ o”   one after another for three times. In the test data, voice samples from 1 to 3 correspond to vowel “ a”  , and voice samples from 4 to 6 correspond to vowel “ o” . The duration of the disease for PD patients in the training dataset ranges from 0 to 6 years. The age range of the patients in the training dataset is from 43 to 77 ($\mu =$ 64.86, $\sigma =$ 8.97). The age range of the the healthy subjects in the training dataset is from 45 to 83 ($\mu =$ 62.55, $\sigma =$ 10.79). The duration of the disease for PD patients in the testing dataset ranges from 0 to 13 years. The age range of the the patients in the testing dataset is from 39 to 79 ($\mu =$ 62.67, $\sigma =$ 10.96). Moreover, the authors of dataset provided Hoehn and Yahr (H &Y) scores for PD patients. The H &Y score provides information about the stage of the disease and its value ranges between 1 and 5^10^. The authors of the second dataset provided Unified Parkinson’s Disease Rating Scale Part III (UPDRS-III) score for the PD patients in the training dataset only. UPDRS III i.e. motor UPDRS ranges from 0 to 108, where 0 represents symptom free and 108 represents severe motor impairments^35,36^. The scores for PD patients are reported in Table 2. For the healthy subjects, UPDRS-III and H &Y values are denoted by n/a. Samuel et al.^37^ suggested that to test the effectiveness of a newly developed machine learning method, it is a good approach to choose dataset(s) that have been extensively tested. Thus, our choice of datasets in this paper was based on the facts discussed in^37^.

**Table 1:** Description of the datasets.

*Code* and Dataset 1 Features	*Code* and Dataset 2 Features	$p_{1}$	$p_{1}$
$F_{11}$ MDVP:Fo(Hz)	$F_{21}$ Jitter (local)	3.0318e$-$05	0.0003
$F_{12}$ MDVP:Fhi(Hz)	$F_{22}$Jitter(local, absolute)	0.00027804	0.009
$F_{13}$ MDVP:Flo(Hz)	$F_{23}$ Jitter (rap)	4.1249$-$05	0.007
$F_{14}$ MDVP:Jitter(%)	$F_{24}$ Jitter (ppq5)	7.8291$-$09	0.005
$F_{15}$ MDVP:Jitter(Abs)	$F_{25}$ Jitter (ddp)	1.2639$-$09	0.007
$F_{16}$ MDVP:RAP	$F_{26}$ Number of pulses	8.6139$-$09	0.006
$F_{17}$ MDVP:	$F_{27}$ Number of periods	2.3799$-$09	< 0.001
$F_{18}$ Jitter:DDP	$F_{28}$ Mean Period	8.1089$-$09	0.039
$F_{19}$ MDVP:Shimmer	$F_{19}$ Standard Dev. Of period	4.1873$-$09	0.007
$F_{110}$ MDVP:Shimmer(dB)	$F_{210}$ Shimmer (local)	3.1154$-$09	0.001
$F_{111}$ Shimmer:APQ3	$F_{211}$ Shimmer (local, dB)	1.1815$-$07	0.039
$F_{112}$ Shimmer:APQ5	$F_{212}$ Shimmer (apq3)	2.0228$-$08	0.001
$F_{113}$ MDVP:APQ	$F_{213}$ Shimmer (apq5)	1.2564$-$11	< 0.001
$F_{114}$ Shimmer:DDA	$F_{214}$ Shimmer (apq11)	1.2008$-$07	0.013
$F_{115}$ Noise-to-Harmonics Ratio	$F_{215}$ Shimmer (dda)	1.3644$-$08	0.968
$F_{116}$ Harmonics-to-Noise Ratio	$F_{216}$ Fraction of locally unvoiced frames	7.5843$-$07	0.928
$F_{117}$ Status (Not feature but label)	$F_{217}$ Number of voice breaks	1.6581$-$05	< 0.001
$F_{118}$ Recurrence Period Density Entropy	$F_{218}$ Degree of voice breaks	0.0018	0.872
$F_{119}$ Detrended Fluctuation Analysis	$F_{219}$ Median pitch	1.5732$-$16	0.050
$F_{120}$ Spread1	$F_{220}$ Mean pitch	7.0891$-$11	< 0.001
$F_{121}$ Spread2	$F_{221}$ Standard deviation	2.9428$-$06	< 0.001
$F_{122}$ D2(Correlation Dimension)	$F_{222}$ Minimum pitch	1.5732$-$16	0.958
$F_{123}$ Pitch Period Entropy	$F_{223}$ Maximum pitch		< 0.001
–	$F_{224}$ Autocorrelation		< 0.001
–	$F_{225}$ Noise-to-Harmonic		0.229
–	$F_{226}$ Harmonic-to-Noise		0.234

*MDVP* Multidimensional voice program, *RAP* Relative amplitude perturbation, *PPQ* Period perturbation quotient, *DDP* Average absolute difference of differences between cycles, divided by the average period, *DDA* The average absolute difference between consecutive differences and the amplitudes of consecutive periods, *APQ3* Three-point amplitude perturbation quotient, *APQ5* Five-point amplitude perturbation quotient, APQ: 11-point amplitude perturbation quotient.$F_{ijk}$: index *i* denotes the dataset number, and *jk* denotes the feature number.

**Table 2:** Details of H &Y scores for PD patients in the first dataset and UPDRS III scores for PD patients in the second dataset.

Subject ID	H &Y	UPDRS	Subject ID	H &Y	UPDRS
1	3.0	23	13	1.0	23
2	2.5	8	14	1.5	5
3	1.5	40	15	2.5	31
4	3	5	16	1.0	55
5	2.5	16	17	4.0	5
6	2.0	46	18	3.0	32
7	2.0	40	19	2.5	26
8	2.0	20	20	2.5	46
9	2.0	11	21	2.5	n/a
10	1.0	12	22	3.0	n/a
11	2.0	24	23	2.5	n/a
12	1.5	32	24	n/a	n/a

### The proposed cascaded system based on $L_{1}$ SVM and DNN

We propose a two-stage feature selection and classification method to detect PD using replicated voice data and various voice records. With the proposed two-stage approach, the time complexity of the predictive model can be reduced. The accuracy can also be improved by eliminating irrelevant features from the feature space. The model that we used for feature refinement is the $L_{1}$-regularized linear SVM, while for classification DNN with optimized hyper-parameters has been used. The models’ formulations, potentially associated problems, and proposed solutions are stated as follows.

For a given dataset *D* with *q* instances: $D = \{(x_{i}, y_{i})|x_{i}\in R^{p}, y_{i}\in \{-1,1\} \}_{i=1}^{q}$ where $x_{i}$ is *i*-th instance and each instance has *p* dimensions or features. And $y_{i}$ denotes class label which may be $-1$ or 1 for binary classification. For the classification problem, SVM learns the hyper-plane given by $wx=b$, where *b* is the bias and *w* is the weight vector. The hyper-plane maximizes the margin distance ${2}/{\parallel {w}\parallel _{2}^2}$.

The primal form of the SVM can be formulated as follows:

1 $$\begin{aligned} \min _{w,b} \frac{1}{2}\parallel {w}\parallel _{2}^2, ~~\text {s. t.}~~ \{y_{i}(wx_{i}+b)\ge 1, i=1,\cdots ,q\} \end{aligned}$$

In 1995, Cortes and Vapnik proposed a modified version of SVM called Soft Margin SVM, which allows for mislabeled instances^38^, and it has the following form:

2 $$\begin{aligned} \min _{w,b,\xi }\underbrace{ \frac{1}{2}\parallel {w}\parallel _{2}^2}_\text {Regularizer} +C\underbrace{\sum _{i=1}^{q}\xi _{i}}_\text {Loss} ~~\text {s. t.} {\left\{ \begin{array}{ll} y_{i}(wx_{i}+b)\ge 1-\xi _{i}, \\ \xi _{i}\ge 0, i = 1,\cdots ,q \end{array}\right. } \end{aligned}$$

where the regularizer or penalty function is $L_{2}$-norm, $C>0$ is the error penalty parameter and $\xi$ is slack variable used for misclassification measurement.

In 1998, Bradley and Mangasarian proposed to use $L_{1}$-norm as the regularizer^39^, and the feature selection can be made using $L_{1}$-norm SVM due to its sparse solutions. It is formulated as:

3 $$\begin{aligned} \min _{w,b,\xi }\underbrace{ \parallel {w}\parallel _{1}}_\text {Regularizer} + C\underbrace{\sum _{i=1}^{q}\xi _{i}}_\text {Loss} ~~\text {s. t. } {\left\{ \begin{array}{ll} y_{i}(wx_{i}+b)\ge 1-\xi _{i}, \\ \xi _{i}\ge 0, i = 1,\ldots ,q \end{array}\right. } \end{aligned}$$

where the regularizer or penalty function is $L_{1}$-norm, $C>0$ is the error penalty parameter and $\xi$ is slack variable used for misclassification measurement. As discussed above, in (3), *w* is the weight vector. changing values of hyper-parameter *C*, different coefficients of *w* shrink towards zero. In fact, with sufficiently small *C*, several fitted coefficients would be exactly zero, i.e., sparse solution. Therefore, $L_{1}$-norm regularization has an inherent feature selection property, i.e., those features whose corresponding coefficients are fitted to zero can be eliminated. Furthermore, as *C* changes, several fitted coefficients will become zero, which will result in different feature subsets^40^. Thus, the optimal subset of features can be obtained by tuning the hyper-parameter *C*. For this purpose, we use HGSA in this paper which will automatically tune the *C* hyper-parameter of the linear SVM model and search the optimal subset of features.

It is worth noting that DNN can extract features by itself. DNNs, including the one used in this paper, use feature extraction rather than feature selection to extract underlined features or rules from the data^32^. We consider only the most important features in feature selection by eliminating the irrelevant features from the feature space. While in feature extraction, all the features are considered, and new ones are extracted. DNNs use a large number of non-linear elements, i.e., neurons, to learn relationships or functions of high complexity. More likely, irrelevant features present in the feature space are also modeled accordingly. Noise is the result of Modeling irrelevant features^32^. Thus, learning the noise from these irrelevant features negatively affects the acquired knowledge of data about the overall distribution of the data^32^. If feature space contains irrelevant features, overfitting the network to the training data is another problem^32,41^. That is when the network learns irrelevant details from the training data. It shows good performance on the training data as it becomes more biased to the previously seen data^42^. But, it fails to generalize to the unseen validation or testing data.

To solve these problems posed by irrelevant features in the feature space, we use $L_{1}$ regularized SVM to make the feature space free from irrelevant features before applying the feature vector to DNN. The SVM model eliminates irrelevant features. To validate the fact that feature selection coupled with the feature extraction capability of DNN improves the performance of DNN, in Section “Comparative study”, we performed experiments by applying all the features to DNN, i.e., removing the feature selection SVM model and then compared it with the proposed $L_{1}$SVM-DNN. The accuracy of 96.87 and 62.5% is obtained for datasets 1 and 2, respectively, when all features were applied to DNN. While accuracies of 100% and 97.5% are obtained for datasets 1 and 2, respectively, using the $L_{1}$SVM-DNN model. Hence, simulation results show that the feature selection capability of the SVM model, when combined with the feature extraction capability of the DNN model, improves the performance of DNN for PD detection problems. HGSA is used to search for advanced or optimal features and is given to a DNN model for classification.

For the given *m* training samples, a DNN models a hypothesis function $h_{\theta }({\textbf{x}})$ parameterized by DNN parameters $\theta \in {\mathbb {R}}^{d}$ where *d* denotes the dimension of $\theta$ and the input feature vector is represented by ${\textbf{x}}$. The $h_{\theta }({\textbf{x}})$ tries to anticipate label $\hat{{\textbf{y}}}$ for input feature vector ${\textbf{x}}$. The aim is to locate those optimum values of $\theta$ for which objective function is minimized as:

4 $$\begin{aligned} J({{\varvec{\theta }}}) = \frac{1}{m} \sum _{j=1}^{m} {\text {cost}}(h_{{\varvec{\theta }}}({\textbf{x}}^{(j)}), {\textbf{y}}^{j}) \end{aligned}$$

We used the ADAM learning algorithm to minimize(4). In this paper, we used default values for hyper-parameters of the ADAM algorithm, i.e., the value of 0.9 for $\beta _{1}$, 0.999 for $\beta _{2}$ and $10^{-8}$ for $\varepsilon$. After optimizing the parameters or weights of the DNN model by ADAM for training data samples, the model performance is evaluated by applying testing data samples. The generalization performance (in terms of % of falsely predicted testing samples), represented by generalization error $\eta$ or validation loss ${\mathcal {L}}(A_{\lambda }, D_\text {train}, D_\text {valid})$. In the expression, $A_\lambda$ denotes the model, $D_\text {valid}$ denotes data on which the loss is evaluated, and $D_\text {train}$ denotes the data on which the model is trained. Our objective is to find $A_\lambda$ that minimizes the validation loss. The hyper-parameter optimization problem under k-fold CV is then to minimize the black box function given as follows:

5 $$\begin{aligned} g(\lambda ) = \frac{1}{k} \sum _{i=1}^{k}{\mathcal {L}}(A_{\lambda }, D^{i}_\text {train}, D^{i}_\text {valid}) \end{aligned}$$

where $\lambda$ denotes the hyper-parameters of DNN and $A_{\lambda }$ represents DNN configuration under $\lambda$ hyper-parameters choice or setting. In order to obtain good performance, optimal hyper-parameters of DNN need to be searched that can lessen the validation loss. Hence, two optimization problems are dealt with here, i.e., searching the optimal value of the hyper-parameter of the SVM model that will yield the optimal subset of features and searching optimal hyper-parameters of the DNN model. In this paper, two optimization problems are merged into one by merging the hyperparameters of the two models. Thus, after merging the two optimization problems into one, (5) can be formulated as:

6 $$\begin{aligned} g(C, \lambda ) = \frac{1}{k} \sum _{i=1}^{k}{\mathcal {L}}(C, A_{\lambda }, D^{i}_\text {train}, D^{i}_\text {valid}) \end{aligned}$$

The minimization of (6) will result in us optimized forms of two models. The merging of hyper-parameters of the two models yields a hybrid grid. Each point on the grid has several coordinates. The first coordinate of each point on the hybrid grid is *C*, i.e., the SVM model’s hyperparameters, while other coordinates are the hyperparameters of the DNN model. The hyper-parameters of the second model contain the number of layers of DNN denoted by *L*, the number of neurons in each hidden layer characterized by $N_{h}$, where *h* indicates the hidden layer number and dropout regularization. Dropout regularization is considered only in those cases when the model is overfitting. To solve the minimization of (6), we use HGSA. Algorithm 1 gives the detailed procedure of the HGSA algorithm.

**Algorithm 1:**
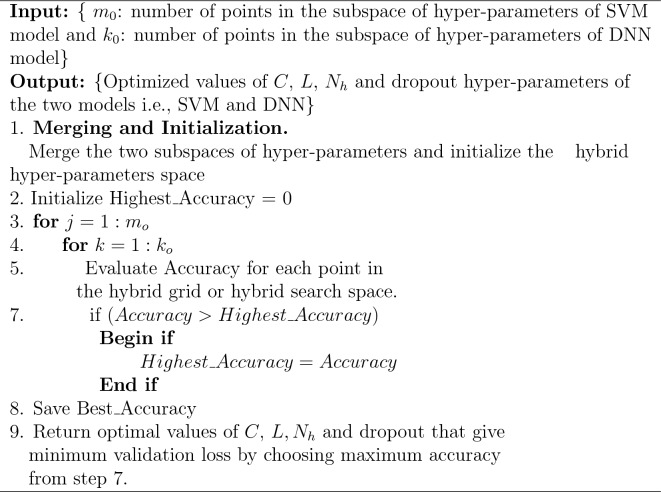
Hyper-parameters optimization of the proposed cascaded system using Hybrid Grid Search Algorithm (HGSA).

### Ethical approval

This article does not contain any studies with human participants or animals performed by any authors.

### Informed consent

Informed consent is not applicable. The study used two publically available datasets^33,34^.

## Results and discussion

For evaluation purposes, both types of cross-validation schemes are utilized, i.e., LOSO CV and k-fold CV with data translation. LOSO CV and k-fold are two widely adopted validation approaches in data analysis. In LOSO CV, the dataset is initially partitioned into $S_{n}$ parts, where $S_{n}$ represents the total number of subjects or individuals.. In each iteration of LOSO CV, the data corresponding to one subject, starting with $S_{1}$, is reserved for testing, while the data from the remaining subjects are utilized for training the model. Similarly, in k-fold CV, the dataset is divided into k subsets or folds. During the first iteration of k-fold CV, the data in the first fold $k=1$ is set aside for testing, while the data from the other folds are employed for model training. In subsequent iterations, the testing fold shifts to the next one $k=2$, and the remaining data continue to serve as the training set. This cycle repeats until all the folds have been used for testing.

**Figure 1:**
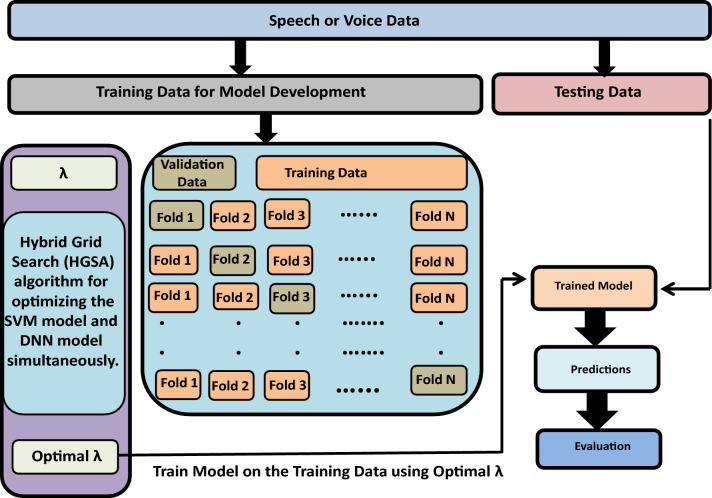
Experimental setup showing model development and testing.

For more practical validation, we carried out model development in phase 1 and model testing in phase 2 as can be seen in Fig. 1. The software package used for these experiments was Python. In all the experiments, *N*1 and $N_{2}$ represent the number of neurons in hidden layer 1 and hidden layer 2 of the network, respectively. While *L* denotes the total number of layers in the neural network and $N_{h}$ represents the number of neurons in each hidden layer when we are using the equal number of neurons in all hidden layers. The learning algorithm used is ADAM. Furthermore, *C* represents the hyper-parameter of the linear SVM model and n denotes the number of features produced by the SVM model. The initial range for the hyperparameters *N*1, $N_{2}$, $N_{h}$ is set between 5 and 100. Likewise, the initial range of the hyperparameters *L* is established between 4 and 10, while the hyperparameter *C* takes an initial range spanning from 0.00001 to 1000.

### Simulation results of dataset 1

#### LOSO cross-validation

In this experiment, LOSO CV is performed on the first dataset. Despite the fact that LOSO CV is the most practical validation scheme for replicated voice data and multiple types of voice data, LOSO CV was ignored in previous studies except^43^ for this dataset. The best results of 100% were obtained for C = 0.5, resulting in a subset of features having only eight features. Moreover, the best result was obtained for optimally configured DNN with five layers i.e. $L=5$, and 20 and 30 neurons in each hidden layer. The same results are also obtained for $L=4$ and $N_{h}=30$. That is, the proposed approach can classify subjects as PD and healthy with an accuracy of 100%. The results of the experiment are reported in Table 3. In the table, the optimal subset of features for n = 8 contains $F_{1}, F_{2}, F_{3}, F_{10}, F_{16}, F_{18}, F_{19}$ and $F_{21}$. It is evident from the table that if optimal hyper-parameters of the DNN model are not utilized, we may obtain poor performance with an optimal subset of features. Thus, better performance can be achieved if extracted features are refined and optimally configured DNN is utilized.

As discussed earlier, the first dataset has the problem of imbalanced classes. The problem of imbalanced classes in data affects the performance of predictive models because the predictive models trained on imbalanced data are more sensitive to detecting the majority class and less sensitive to the minority class^44^. Thus, there is a need to balance the training process of the predictive model. There are two ways-Under-sampling the majority class and over-sampling the minority class. Over-sampling is very easy for image datasets because, with simple operations like rotations and translation, we can easily over-sample the minority class. For voice data, we have used the under-sampling method. However, in literature, more advanced techniques used for under-sampling did not significantly improve simply selecting random samples. Hence, in this paper, we performed random under-sampling during the training process.

The practical demonstration of the problems posed by imbalance classes is given in Table 3. The last three rows of the table, separated by a horizontal line, are the results obtained when no measure is taken to balance the training process. The simulation results show that the model fails to perform better even with optimally configured DNN and the optimal subset of features. The reason is that machine learning models are sensitive to detecting the majority class and less susceptible to detecting the minority when imbalanced classes are used to train the model. That is why in the last three rows, the model results in poor specificity. Thus, it is of paramount importance to balance classes during the training process.

**Table 3:** Results of LOSO cross-validation for dataset 1.

Hyperparameters				Evaluation Metrics			
C	n	L	$\text {N}_\text {h}$	ACC (%)	Sen. ( %)	Spec. (%)	MCC
0.5	8	4	10	93.75	91.66	100	0.856
0.5	8	4	20	90.62	87.50	100	0.797
**0.5**	**8**	**4**	**30**	**100.0**	**100.0**	**100**	**1.000**
0.5	8	5	10	96.87	95.83	100	0.922
**0.5**	**8**	**5**	**20**	**100.0**	**100.0**	**100**	**1.000**
**0.5**	**8**	**5**	**30**	**100.0**	**100.0**	**100**	**1.000**
0.5	8	4	30	87.50	100.0	50.0	0.654
0.5	8	5	20	84.37	100.0	37.5	0.557
0.5	8	5	30	87.50	100.0	50.0	0.654

C: Hyper-parameter of the SVM model. n: number of selected features. L: layers in DNN. $\text {N}_\text {h}$: Width of each hidden layer. ACC[URACY]: Percentage of accuracy obtained for LOSO CV, Sen[sitivity], Spec[ificity].

Significant values are in bold.

#### k-fold cross-validation with k=10

The second experiment that is performed on the first dataset is a k-fold CV. The value of k is chosen here to be 10. The results for different hyper-parameter configurations are given in Table 4. HGSA searches for the best accuracy of 100% for a 10-fold CV. The achieved accuracy via 10-fold CV is the same as the accuracy achieved in^45^. In^45^, the 10-fold experiment was also conducted on the second dataset and achieved 90% accuracy. Our proposed model achieved 97.5% for 10-fold CV on the second dataset, which proves the effectiveness of the proposed diagnostic system. The optimal subset of features with $n=1$ contains $F_{2}$ and with n = 7 contains $F_{1}, F_{2}, F_{3}, F_{10}, F_{16}, F_{19}$ and $F_{21}$.

**Table 4:** Results of 10-fold CV for dataset 1.

Hyperparameters				Evaluation Metrics			
C	n	$N_{1}$	$N_{2}$	ACC (%)	Sens. (%)	Spec. (%)	MCC
0.001	1	5	5	12.50	0.000	50.00	$-$ 0.625
**0.001**	**1**	**10**	**11**	**100.0**	**100.0**	**100.0**	**1.000**
3.000	7	5	5	50.00	45.83	62.50	0.072
**3.000**	**7**	**12**	**2**	**96.87**	**95.83**	**100.0**	**0.922**
45.00	10	27	27	59.37	62.50	50.00	0.110
**45.00**	**10**	**30**	**27**	**96.87**	**95.83**	**100.0**	**0.922**

C: Hyper-parameter of the SVM model. n: number of selected features. $N_{1}$: Width of first hidden layer. $N_{1}$: Width of second hidden layer. ACC[URACY]: Percentage of accuracy obtained for 10fold CV, Sen[sitivity], Spec[ificity].

Significant values are in bold.

### Simulation results of dataset 2

#### LOSO cross validation on training database

In this experiment, LOSO CV is performed on the training database of the second dataset. We achieved state-of-the-art results with an accuracy of 100%, which is the highest classification accuracy reported so far for LOSO CV on the training database. The results of the experiment are given in Table 5. The proposed approach has the capability to classify subjects as PD and healthy with an accuracy of 100%. The best results are obtained for C hyper-parameter equal to 0.0015 for this dataset, resulting in a feature subset consisting of only seven features. It is important to note that 100% result for LOSO CV does not mean that the proposed system can correctly classify all samples of the dataset. Because a subject is classified as PD if more than half of its samples are predicted as 1, otherwise the subject is classified as healthy. Thus, it is expected that for any disease having more than one sample per patient, the proposed system could be an ideal candidate for diagnosis. Moreover, optimal subset of features for C = 0.0015 and with n = 7 contains $F_{5}, F_{10}, F_{15}, F_{19}, F_{21}, F_{24}$ and $F_{26}$. Additionally, the best result of 100 % was obtained for optimally configured DNN with five layers i.e. L = 5 and 30 neurons in each hidden layer. It is evident from Table 5 that if optimal hyperparameters of the DNN model are not utilized, we may obtain poor performance with an optimal subset of features. Thus, better performance can be achieved if extracted features are refined and optimally configured DNN is utilized.

**Table 5:** Results of LOSO on train database of dataset 2.

Hyperparameters				Evaluation Metrics			
C	n	L	$\text {N}_\text {h}$	ACC (%)	Sen. ( %)	Spec. (%)	MCC
0.0015	7	4	20	95.0	100	90.0	0.904
0.0015	7	4	30	97.5	100	95.0	0.951
0.0015	7	4	40	97.5	100	95.0	0.951
**0.0015**	**7**	**5**	**20**	**95.0**	**100**	**90.0**	**0.904**
**0.0015**	**7**	**5**	**30**	**100**	**100**	**100**	**1.000**
0.0015	7	5	40	95.0	100	90.0	0.904

C: Hyper-parameter of the SVM model. n: number of selected features. L: layers in DNN. N_h_: Width of each hidden layer. ACC[URACY]: Percentage of accuracy obtained for LOSO CV on training database, Sen[sitivity], Spec[ificity].

Significant values are in bold.

#### LOSO cross-validation on testing database

In this experiment, LOSO CV is performed on the testing database of the second dataset. This dataset is an independent dataset collected from new 28 patients under the same conditions in which the training dataset was collected. This dataset aims to validate the performance of the proposed system achieved on the training dataset. Since this data only contain patient subjects and no healthy subject, thus its specificity cannot be reported. The DNN model is trained on a train data file, but it is transformed into a new dataset by extracting only those concerned with vowel phonations. The main reason for creating modified train data is that the test data, in this case, contains only vowel phonations. The simulation results for this experiment are given in Table 6. From the results, it is clear that maximum accuracy of 78.57% is obtained. It is due to the overfitting of the model to the training data. Thus to avoid the model from overfitting, we bring into account dropout regularization. With 0.3 dropouts, the proposed method achieved an accuracy of 100%. The dropout regularization is applied to hidden layers of the DNN model. Dropout is a hyperparameter that is used when the DNN is facing the problem of overfitting. It is important to note that according to the proper unbiased validation approach depicted in Fig. 1, the accuracy on the testing dataset should be reported 96.42% not 100% because during the model development phase (results given in Table 5), the optimal model is produced under hyperparameters configuration of $n=7$, $L=5$ and $N_{h}=30$.

**Table 6:** LOSO CV on a test database of dataset no 2.

Hyperparameters		Evaluation Metrics				
C	n	L	$\text {N}_\text {h}$	Dropout	ACC (%)	Sens. (%)
0.0015	7	4	10	–	78.57	78.57
0.0015	7	4	20	–	75.00	75.00
0.0015	7	4	30	–	64.28	64.28
0.0015	7	4	40	–	75.00	75.00
0.0015	7	5	10	–	67.85	67.85
0.0015	7	5	20	–	75.00	75.00
0.0015	7	5	30	–	71.42	71.42
0.0015	7	5	40	–	75.00	75.00
0.0015	7	5	10	0.3	92.85	92.85
**0.0015**	**7**	**5**	**20**	**0.3**	**100.0**	**100.0**
0.0015	7	5	30	0.3	96.42	96.42
0.0015	7	5	40	0.3	92.82	92.82

C: Hyper-parameter of the SVM model. n: number of selected features. L: layers in DNN. N_h_: Width of each hidden layer. Dropout: A hyper-parameter utilized when the network is over-fitting. ACC[URACY], Sen[sitivity].

Significant values are in bold.

#### k-fold cross validation with k = 10 on training data of dataset 2

The results of the 10-fold CV experiment for dataset 2 are given in Table 7. It is important to note that so far the highest accuracy achieved for 10-fold CV is 90% (see Table 11). The proposed diagnostic system achieved the best PD detection accuracy of 97.5 %. The obtained accuracy is the highest accuracy for k-fold cross-validation for this dataset. Moreover, the optimal subset of features obtained at $C=0.001$ and with $n=1$ contains $F_{19}$ while the optimal subset of features with at $C=0.01$ and with $n=4$ contains $F_{10}, F_{18}, F_{19}$ and $F_{21}$.

**Table 7:** Results of 10-fold on train data file of dataset 2.

Hyperparameters				Evaluation Metrics			
C	n	${\textbf{N}}_{\textbf{1}}$	${\textbf{N}}_{\textbf{2}}$	ACC (%)	Sen. ( %)	Spec. (%)	MCC
0.001	1	5	5	37.5	0.00	75.0	–0.377
0.001	1	11	11	12.5	00.0	25.0	–0.774
0.001	1	22	22	5.00	10.0	0.00	–0.904
0.001	1	28	28	70.0	60.0	80.0	–0.408
**0.001**	**1**	**28**	**11**	**97.5**	**95.0**	**100**	**0.951**
0.001	1	30	30	50.0	40.0	60.0	0.000
**0.010**	**4**	**55**	**51**	**95.0**	**90.0**	**100**	**0.904**

C: Hyper-parameter of the SVM model. n: number of selected features. $N_{1}$: Width of first hidden layer. $N_{1}$: Width of second hidden layer. ACC[URACY], Sen[sitivity], Spec[ificity].

Significant values are in bold.

## Comparative study

In this section, the performance of the proposed method is compared with other well-known machine learning models and with previously published work that used the two benchmark voice datasets.

### Comparison of the proposed method with other models for dataset 1

For validation purposes, we also carried out experiments by cascading the features refinement model i.e. $L_{1}$ SVM with other renowned classifiers namely SVM and artificial neural network (ANN) owing to their remarkable performance on many other biomedical problems. Furthermore, we also checked the performance of the conventional DNN model without any feature refinement module. Next, we developed three similar hybrid systems i.e., SVM-SVM(Lin) and SVM-SVM(RBF), and SVM-ANN, where the first SVM model is $L_{1}$ regularized linear SVM model that is used for features refinement while the second model is used as a predictive model. In the case of the SVM-SVM hybrid model, we denote the hyper-parameter of the feature selection model by $C_{1}$ while the hyper-parameter of the predictive SVM model by $C_{2}$. In addition, g denotes the gamma hyperparameter of the SVM predictive model when it uses the RBF kernel. All these experiments were performed using a 10-fold CV. The goal is to evaluate the feature refinement capabilities of the $L_{1}$ SVM when it is cascaded state-of-the-art classifiers. Furthermore, all the cascaded models were optimized by using the HGSA approach. The results are tabulated in Table 8.

**Table 8:** Results of other models on dataset 1.

Hyperparameters						Evaluation Metrics		
Method	${\textbf{C}}_{\textbf{2}}/{\textbf{N}}_{\textbf{1}}$	${\textbf{g}}/{\textbf{N}}_{\textbf{2}}$	${\textbf{C}}_{\textbf{1}}$	**n**	ACC (%)	Sen. ( %)	Spec. (%)	MCC
ANN	25	–	–	22	84.37	91.66	62.50	0.567
SVM-ANN	28	–	3900	18	87.50	83.33	100.0	0.745
SVM(Lin)	0.001	–	–	22	34.37	33.33	37.50	–0.257
SVM-SVM(Lin)	0.015	–	0.001	1	65.62	87.5	0.000	–0.185
SVM(RBF)	0.025	0.0005	–	22	78.12	79.16	75.00	0.493
SVM-SVM(RBF)	0.4	0.001	0.001	1	84.37	91.66	62.50	0.567
DNN	26	1	–	22	96.87	95.83	100.0	0.922
**Proposed**	**10**	**11**	**0.001**	**1**	**100.0**	**100.0**	**100.0**	**1.000**

$C_{2}$/$N_{1}$: Hyper-parameter of SVM predictive model or width of first hidden layer in case of ANN or DNN predictive model. g/$N_{2}$: g hyper-parameter of SVM predictive model or width of second hidden layer for DNN predictive model. $C_{1}$: Hyper-parameter of the $L_{1}$ regularized SVM. n: the size of the optimal subset of features.

Significant values are in bold.

### Comparison of the proposed method with other models for dataset 2

The same types of cascaded models were also developed for the second dataset. The results are reported in Table 9. From Tables 8 and 9, it is clear that the proposed method shows better performance. Additionally, in each case, the $L_{1}$ SVM produces features of better quality, and hence performance of the predictive model is improved whether it is SVM, ANN, or DNN. Thus, these results validate the feature refinement capabilities of the developed cascaded systems.

**Table 9:** Results of other models on dataset 2.

Hyperparameters						Evaluation Metrics		
Method	${\textbf{C}}_{\textbf{2}}/{\textbf{N}}_{\textbf{1}}$	${\textbf{g}}/{\textbf{N}}_{\textbf{2}}$	${\textbf{C}}_{\textbf{1}}$	n	ACC (%)	Sen. ( %)	Spec. (%)	MCC
ANN	15	–	–	26	62.5	60	65.0	0.250
SVM-ANN	6	–	0.005	2	67.5	50	85.0	0.373
SVM(Lin)	0.0003	–	–	26	45.0	50	40.0	–0.100
SVM-SVM(Lin)	0.01	–	0.001	1	90.0	80	100	0.816
SVM(RBF)	50	0.0001	–	26	45.0	45	45.0	–0.100
SVM-SVM(RBF)	30	0.045	0.001	1	60.0	60	60.0	0.200
DNN	34	34	–	26	62.5	55	70.0	0.252
**Proposed**	**28**	**11**	**0.001**	**1**	**97.5**	**95**	**100**	**0.951**

$C_{2}$/$N_{1}$: Hyper-parameter of SVM predictive model or width of first hidden layer in case of ANN or DNN predictive model. g/$N_{2}$: g hyper-parameter of SVM predictive model or width of second hidden layer for DNN predictive model. $C_{1}$: Hyper-parameter of the $L_{1}$ regularized SVM. n: the size of an optimal subset of features.

Significant values are in bold.

### Comparison with previously reported methods

For comparison purposes, Tables 10 and 11 list accuracies obtained in previous studies by different methods applied to the two voice recording-based PD datasets. As shown in these tables, our developed model can yield better classification accuracy than previously proposed methods in the literature.

**Table 10:** Performance of different methods recently published for dataset 1.

Reference of Study	Method	Accuracy (%)
^10^	feature selection (FS) integration with SVM	91.4
^22^	Neural Network	92.9
^43^	SVM integrated with FS	92.75
^46^	modified FS	89.47
^47^	Random Forest (RF) based ensemble	87.1
^48^	Integration of feature extraction (FE) with SVM	93.47
^49^	Similarity classifier integrated with SVM	85.03
^50^	Heuristic algorithms based FS	84.01
^23^	ensemble of neural networks	91.20
^51^	Fuzzy kNN (f-kNN)	96.07
^52^	Adaptive f-kNN	97.47
^53^	RF + sample selection	87.8
^54^	SVM integrated with FS	90
^19^	Integration of Autoencoders and classifiers	94 to 98
^55^	RF ensemble + FS	97
^56^	SVM with web application	97.1
^45^	Ensembles of NNs	90
^21^	DNN	93.79
^57^	heuristically optimized SVM and RF	97.42
Current	SVM cascaded with DNN model	100 (LOSO CV)
Current	SVM cascaded with DNN model	100 (10-fold CV)

**Table 11:** Performance of different methods recently published for dataset 2.

Reference of Study	Method	Accuracy (%)
^6^	KNN and SVM	68.45
^15^	FS with classification	57.5 , 68.94
^58^	Ensemble approach	74.17
^59^	sample selection and multiple classifiers	87.50
^53^	Sample selection with ensemble approach	81.5 and100
^60^	Feature extraction with HFCC and SVM	87.5 and 100
^61^	feature selection with SVM	82.50
^62^	Trees and RF	66.5
^19^	Autoencoders with classifiers	94.17
^63^	Feature extraction using MFCC and SVM	82.5
^64^	Enemble of NNs	Average 75
^45^	Ensembles of NNs	90
^21^	DNN	68.05
^65^	evolutionary optimized classifiers	83.68
^66^	LDA-GA-NN	82.14
Current	SVM cascaded with DNN model	100 (LOSO on training database)
Current	SVM cascaded with DNN model	96.42 (LOSO on testing database)
Current	SVM cascaded with DNN model	97.50 (10-fold CV)

Based on data in Tables 10 and 11, we are in a position to conclude that our developed diagnostic system gives state-of-the-art performance in terms of PD detection accuracy.

## Limitations of the study

Although this study showed good performance in terms of differentiating PD patients from healthy subjects, there are some limitations. One limitation pertains to the data used in the study. Information such as the severity of the disease in PD patients from the testing dataset of the second dataset and whether the data collection was carried out in the ON or OFF state of the disease is missing. The study did not investigate whether accuracy varies depending on disease duration and severity. Another diagnostic challenge in Parkinsonism is differentiating between idiopathic PD and atypical PD (e.g., progressive supranuclear palsy (PSP), multiple system atrophy (MSA), corticobasal syndrome (CBS), Dementia with Lewy Bodies (DLB)), where vocal dysfunction is also manifested^67^. The study did not investigate this kind of differential diagnosis.

## Conclusion

This paper has addressed two primary issues concerning the automated detection of PD. Firstly, it has highlighted the inadequacies of validation methodologies employed in previous studies, which led to the creation of biased predictive models. Secondly, it has recognized the persistent challenge of achieving high PD detection rates when unbiased models are employed. To mitigate bias, this study has adopted appropriate validation approaches. In addition, to enhance the accuracy of PD detection, a two-stage diagnostic system, referred to as $L_{1}$SVM-DNN, has been proposed. Notably, unlike previous methods, this research has emphasized the independence of model development and testing phases. Two benchmark datasets were employed for validation purposes. The experimental results have demonstrated that the proposed method attains a classification accuracy of 97.5% with 10-fold CV and an impressive 100% accuracy with LOSO CV. For generalization purposes, we also evaluated the optimally developed model on testing dataset and obtained 96.42% accuracy. Based on these outcomes, it can be confidently asserted that the developed cascaded system holds significant promise in automated differentiation of PD patients from healthy subjects.

Although the $L_{1}$SVM-DNN approach showed outstanding performance in terms of differentiating PD patients from healthy subjects, from a clinical diagnostic perspective, this kind of automated differentiation has limited significance. This is because, in real-time applications, differentiating between idiopathic PD and atypical PD (e.g., PSP, MSA, CBS, DLB), where vocal dysfunction is also manifested, is a more challenging task. Therefore, future efforts should focus on the collection of a multi-class dataset, including data from healthy subjects, idiopathic PD, and atypical PD and its subtypes. Unbiased machine learning models, like $L_{1}$SVM-DNN, should be trained and tested on such multi-class problems. These models would have more significance and could be deployed in hospitals and clinics for real-time diagnostic applications.

## Data Availability

The datasets analyzed during the current study are available in the UCI Machine Learning Repository, https://doi.org/10.24432/C5NC8M, and https://doi.org/10.24432/C59C74.
