# Author Correction: Parkinson’s disease detection based on features refinement through L1 regularized SVM and deep neural network

**DOI:** 10.1038/s41598-024-74500-7

**Published:** 2024-10-09

**Authors:** Liaqat Ali, Ashir Javeed, Adeeb Noor, Hafz Tayyab Rauf, Seifedine Kadry, Amir H. Gandomi

**Affiliations:** 1 Department of Electrical Engineering, University of Science and Technology Bannu, Bannu, Pakistan; 2 Aging Research Center, Karolinska Institutet, Solna, Sweden; 3 Department of Information Technology, Faculty of Computing and Information Technology, King Abdulaziz University, 80221 Jeddah, Saudi Arabia; 4 Bool Mind Software Technologies, Mequon, WI 53092 USA; 5 Department of Applied Data Science, Norof University College, Kristiansand, Norway; 6 Artifcial Intelligence Research Center (AIRC), Ajman University, Ajman, 346 United Arab Emirates; 7 Department of Electrical and Computer Engineering, Lebanese American University, Byblos, Lebanon; 8 Faculty of Engineering and Information Technology, University of Technology Sydney, Ultimo, NSW 2007 Australia; 9 University Research and Innovation Center (EKIK), Óbuda University, Budapest, 1034 Hungary

Correction to: *Scientific Reports*10.1038/s41598-024-51600-y, published online 16 January 2024.

In the original version of this Article Hafiz Tayyab Rauf was incorrectly affiliated with ‘Centre for Smart Systems, AI and Cybersecurity, Staffordshire University, Stoke-on-Trent, ST4 2DE, UK’.

The correct affiliation is listed below.

Bool Mind Software Technologies, Mequon, WI, 53092, USA

The original Article has been corrected.

